# NEDD4 promotes reactive astrogliosis by enhancing K63-linked ubiquitination and inhibiting chaperone-mediated autophagy degradation of YAP1

**DOI:** 10.7150/ijbs.123639

**Published:** 2026-01-01

**Authors:** Wu Ye, Yigang Li, Wei Wang, Dishui Pan, Xiaokun Wang, Yao Gu, Yufeng Zhu, Haofan Wang, Xuanyu Lu, Dongdong Jiang, Pengyu Tang, Haoming Shu, Jun Ma, Weihua Cai

**Affiliations:** 1 Department of Orthopedics, The First Affiliated Hospital of Nanjing Medical University, Nanjing, 210029, Jiangsu, China.; 2 Department of Stress Medicine, Faculty of Psychology, Naval Medical University, Shanghai 200433, China.; 3 Department of Orthopedics, Nanjing First Hospital, Nanjing Medical University, Nanjing 210006, China.; 4 Department of Orthopedics, Shanghai General Hospital, Shanghai Jiao Tong University School of Medicine, Shanghai 200080, China.

**Keywords:** reactive astrogliosis, NEDD4, YAP1, ubiquitination, chaperone-mediated autophagy

## Abstract

Following spinal cord injury (SCI), the transcriptional regulator yes-associated protein 1 (YAP1) is upregulated and accumulates in the nuclei of astrocytes, where it promotes reactive astrogliosis—a process that critically influences wound healing and neurological function recovery. However, the mechanisms regulating YAP1 in reactive astrocytes after SCI remain largely unclear. This study, we identify the E3 ubiquitin ligase NEDD4 as a critical regulator of astrocyte reactive proliferation. NEDD4 enhances astrogliosis by suppressing YAP1 degradation. Conditional deletion of *Nedd4* in astrocytes markedly attenuates reactive astrogliosis *in vivo,* and results in heightened inflammation, exacerbated neuronal injury, and impaired functional recovery following SCI. Importantly, YAP1 overexpression is sufficient to reverse the pathological and functional consequences of *Nedd4* deficiency. Mechanistically, NEDD4 interacts with YAP1 and mediates K63-linked ubiquitination at lysine 254, thereby preventing its degradation via the chaperone-mediated autophagy (CMA) pathway involving HSC70. Furthermore, we demonstrate that the ROS-FOXM1 signaling cascade drives NEDD4 expression, thereby stabilizing YAP1 and promoting astrocyte proliferation. In summary, our findings underscore the pivotal role of the ROS-FOXM1-NEDD4-YAP1 signaling cascade in controlling astrocytic activation and tissue regeneration post-SCI, positioning NEDD4 as a viable target to regulate astrogliosis and facilitate neurological restoration after SCI.

## Introduction

Spinal cord injury (SCI) causes profound and often irreversible deficits in motor, sensory, and autonomic functions, largely attributable to the limited regenerative potential of the adult mammalian central nervous system (CNS). Consequently, the resulting disability contributes to significant personal suffering and considerable socioeconomic burden worldwide 1,2. The pathological mechanisms underlying SCI comprise an initial mechanical insult that directly disrupts axons, vasculature, and cellular membranes, followed by a cascade of secondary events—such as spinal cord edema, demyelination, and glial-mediated inflammation—that exacerbate tissue destruction and aggravate neurological deficits 3. Despite the greatly increased life expectancy following SCI, clinical treatment for restoration of function remains limited 4. The delayed nature of secondary injury provides a therapeutic window of opportunity, so it is critical to elucidate the underlying molecular and cellular mechanisms for the development of practical clinical interventions.

As the principal glial component of the CNS, astrocytes orchestrate a range of early responses to SCI, including re-establishment of the blood-spinal cord barrier, regulation of synaptic connectivity and stability, and provision of metabolic and trophic support to surrounding neural cells 5,6. After SCI, astrocytes surrounding the lesion center undergo a specific reactive transformation termed astrogliosis which is typically characterized by cellular hypertrophy, proliferation, and process extension as well as a myriad of gene expression changes linked to both reparative and degenerative processes 7,8. For promotion of repair, these reactive astrocytes establish a dense cellular barrier that isolates the injured area, thereby limiting the infiltration of inflammatory cells, protecting healthy tissue, promoting the efficient induction of repair mechanisms, and ultimately enhancing functional recovery. Accumulating evidence suggest that promotion of reactive astrocyte proliferation is conducive to functional recovery after SCI 9-11.

Yes-associated protein 1 (YAP1) is a transcriptional regulator implicated in cell differentiation and reactive astrogliosis after SCI 12,13. In response to various external and intracellular signals, YAP1 translocate from the cytoplasm into the nucleus where it acts as a transcriptional co-factor binding TEAD and promoting transcription of target genes related to cell proliferation 14,15. YAP1 undergoes extensive post-translational regulation, including phosphorylation, methylation, O-GlcNAcylation, and ubiquitination, which collectively modulate its stability and functional activity 16-19. However, the specific mechanisms regulating YAP1 expression in astrocytes after SCI require further investigation as regulation of astrogliosis is known to influence functional recovery.

Ubiquitination is a major PTM regulating molecular activity and fate. Covalent attachment of ubiquitin to substrate proteins by an E1 ubiquitin activating enzyme, E2 ubiquitin conjugating enzyme, and E3 ubiquitin protein ligase driven by ATP hydrolysis alters various protein-protein interactions 20. These different fates are controlled by the specific pattern of ubiquitination 21. Neural precursor cell expressed developmentally downregulated 4 (NEDD4), also known as NEDD4-1, is a HECT family E3 ligase with critical functions throughout life, including contributions to CNS development and injury repair 22-25. For example, NEDD4 contributes to developmental myelination by stabilizing VHL expression in oligodendrocytes through K63-linked ubiquitination 26, and to neurite development by regulating Rap2A 27. NEDD4 interacts with PTEN within dorsal root ganglion neurons, thereby enhancing intrinsic regenerative signaling and promoting axonal repair after injury 28. However, the role of NEDD4 in reactive astrogliosis following SCI remains poorly understood.

Here, we reveal that the E3 ubiquitin ligase NEDD4 acts as an essential modulator of astrocyte reactivity by stabilizing YAP1 and promoting reactive proliferation. Mechanistically, NEDD4 interacts with YAP1 and mediates K63-linked ubiquitination at lysine 254, thereby preventing its degradation via the chaperone-mediated autophagy (CMA) pathway involving HSC70. Furthermore, the ROS-FOXM1 signaling cascade drives NEDD4 expression, thereby stabilizing YAP1 and promoting astrocyte proliferation. Overall, the ROS-FOXM1-NEDD4-YAP1 axis emerges as a central regulatory pathway orchestrating astrocyte reactivity and tissue regeneration after SCI with NEDD4 representing a potential point of therapeutic modulation to enhance repair processes.

## Materials and Methods

### Animals

*Nedd4^fl/fl^* and *Aldh1l1^CreER^* mice (C57BL/6 background) were obtained from Cyagen Biosciences (Guangzhou, China). *Nedd4^fl/fl^* mice containing LoxP sites flanking exon 9 were bred with *Aldh1l1^CreER^* mice to generate *Nedd4^fl/fl^*-*Aldh1l1^CreER^* and *Nedd4^fl/fl^* littermates for experiments. Mice were housed under standard laboratory conditions with free availability of food and water. All animal procedures were approved by the Ethics Committee of the First Affiliated Hospital of Nanjing Medical University.

### Tamoxifen administration

Tamoxifen (Sigma, T5648, USA) was prepared at a concentration of 15 mg/ml by dissolving it in corn oil (Sigma, C8267, USA). Mice administered intraperitoneal injections of tamoxifen at a dose of 75 μg per gram of body weight on designated days.

### SCI model

The crush model of SCI was created following established procedures described in earlier reports 29. Mice were anesthetized with inhaled isoflurane, and a laminectomy at T9 was performed to expose the dorsal spinal cord. The exposed spinal cord was compressed using 0.2-mm forceps for 2 seconds. Hemostasis was achieved, and the overlying muscles and skin were sutured in layers. Manual bladder expression was carried out twice daily postoperatively until voluntary urination returned. For sham controls, animals underwent laminectomy without subsequent injury.

In the contusion injury model, a 5-g rod was then dropped from a height of 6.5 cm using a spinal cord impactor device (68079, RWD, China) to induce the injury. Postoperative care was identical to that provided in the contusion model.

### Behavioral analysis

To assess hindlimb locomotor function, mice were allowed to walk along a custom-built track measuring 60 cm in length and 4 cm in width. Key anatomical landmarks used for kinematic analysis included the iliac crest, hip, knee, ankle, and distal toe. Continuous locomotor sequences were recorded as each mouse traversed a 30 cm central recording zone, maintaining a straight trajectory without head deviation. Videos were captured using an iPhone camera at 60 frames per second.

Following the recommended procedures, DeepLabCut was trained to automatically identify and track anatomical landmarks, providing the X and Y coordinates of each point throughout all video frames 30. The processed data were subsequently imported into MATLAB (MathWorks, Inc.) for quantitative analysis. Parameters such as maximal toe and iliac crest heights, stride length, and the swing angles of the knee and ankle joints were extracted, and all measurements were performed in a blind fashion. To visualize and interpret hindlimb kinematics more intuitively, simplified skeletal representations were generated in MATLAB by connecting the tracked landmarks. The standard connections included the iliac crest-hip joint, hip joint-knee joint, knee joint-ankle joint, and ankle joint-toe joint.

### Electromyography test

Motor evoked potentials (MEPs) were assessed using electromyography to evaluate functional signal transmission through the spinal cord. Electrical stimulation was delivered through an electrode placed at the rostral portion of the spinal cord, and evoked potentials were recorded via an electrode inserted into the biceps femoris of the hindlimb. The reference electrode was affixed to the distal tendon, and the ground electrode was implanted subcutaneously.

MEPs were elicited by delivering a single square-wave electrical pulse (0.5 mA intensity, 0.5 ms duration, at 1 Hz frequency). The resulting evoked responses were analyzed to determine signal amplitude and latency, which served as indicators of motor pathway conduction.

### Immunofluorescence staining

Mice were perfused transcardially with cold 0.9% saline followed by 4% paraformaldehyde (PFA). The spinal cords were gently dissected, fixed in 4% PFA at 4 °C overnight, and subsequently cryoprotected in 20% and 30% sucrose solutions. Tissues were embedded and sectioned at a thickness of 12 μm using a cryostat. Cryosections were washed with phosphate-buffered saline (PBS) and subsequently incubated at room temperature for 1 hour in a blocking buffer containing 5% bovine serum albumin (BSA) and 0.3% Triton X-100. After incubation overnight at 4 °C with primary antibodies diluted in 5% BSA, the sections were treated with fluorophore-conjugated secondary antibodies (Alexa Fluor 488, 594, or 647; Jackson ImmunoResearch, USA) for 2 hours at room temperature. Nuclear staining was performed with DAPI, and fluorescence images were obtained using a THUNDER DMi8 microscope (Leica Microsystems, Germany). The following primary antibodies were used: Mouse anti-YAP1 (sc-101199, Santa Cruz); Mouse anti-NEDD4 (sc-518160, Santa Cruz); Rabbit anti-NEDD4 (21698-1-AP, Proteintech); Rat anti-IBA1 (ab283346, Abcam); Rabbit anti-NeuN (ab177487, Abcam); Mouse anti-NeuN (ab104224, Abcam); Rabbit anti-GFAP (GB11096, Servicebio); Mouse anti-GFAP (GB12090, Servicebio); CoraLite® Plus 750-conjugated GFAP (CL750-16825, Proteintech); Rabbit anti-CD68 (28058-1-AP, Proteintech); Rabbit anti-Ki67 (GB111499, Servicebio); Rabbit anti-Flag (80010-1-RR, Proteintech).

### Cell lines and primary astrocyte culture

HEK293T cells (Cell Bank of the Chinese Academy of Sciences, Shanghai, China) were cultured in DMEM (Gibco, USA) supplemented with 10% fetal bovine serum and 1% penicillin-streptomycin and grown at 37 °C in a 5% CO_2_ humidified incubator.

Primary astrocytes were isolated from neonatal mouse brains following established protocols. In brief, cerebral tissue was dissected and chopped into 0.5-1.0 mm³ fragments, then digested in 0.125% trypsin-EDTA (Gibco, USA) at 37 °C for 20 minutes, with gentle agitation every 5 minutes. The digested tissue was centrifuged to remove debris, resuspended in DMEM, and filtered through a 100-μm nylon mesh to obtain a single-cell suspension. Cells were seeded into T75 flasks pre-coated with poly-D-lysine and maintained with medium changes every 2 days until confluence was reached. To enrich for astrocytes, the mixed glial cultures were subjected to differential shaking: first at 180 rpm for 30 minutes, followed by 240 rpm for 6 hours to eliminate microglia and oligodendrocyte progenitors. The adherent astrocyte-enriched population was then used for subsequent experiments.

### Astrocyte scratch injury assay *in vitro*

Scratch assays were conducted on primary astrocyte cultures as previously described 10,31. Briefly, mouse astrocytes were plated in six-well plates and cultured until reaching over 90% confluence. A straight scratch was generated across the confluent monolayer with a sterile 200 μL pipette tip. Detached cells and debris were removed by gentle rinsing with PBS, and cultures were subsequently incubated in DMEM for 48 hours. Astrocyte proliferation following injury was evaluated using a 5-ethynyl-2'-deoxyuridine (EdU) incorporation assay kit (Beyotime, China) following the manufacturer's protocol. Proliferating astrocytes were quantified as the percentage of EdU-positive (EdU⁺) cells within the population of GFAP-positive (GFAP⁺) cells. Additionally, the length of astrocytic processes extending into the wound area was measured using ImageJ software (National Institute of Mental Health, USA).

### Plasmids and adenovirus transfection

The shRNA constructs targeting NEDD4, ATG5, BECN1, HSC70, LAMP2A, FOXM1, as well as the non-targeting control (sh-NC), along with a range of expression plasmids—including Myc-tagged NEDD4 (wild-type (WT) and the C854A mutant (MUT) for mouse; WT and C1286A MUT for human), HA-tagged ubiquitin variants (WT and MUTs at K6, K11, K27, K29, K33, K48, and K63), His-tagged HSC70 (WT, Q186A, Q323A, and Q328A MUTs), HA-tagged LAMP2A, and Flag-tagged YAP1 (WT and MUTs at residues 76R, 90R, 97R, 102R, 181R, 204R, 254R, 280R, 315R, 321R, 342R, 494R, and 497R)—were all obtained from Genebay Biotech (Nanjing, China). Primary astrocytes and HEK293T cells were transfected using Lipofectamine 3000 reagent (Thermo Fisher Scientific), according to the manufacturer's protocol.

### Administration of adeno-associated virus (AAV) *in vivo*

To upregulate exogenous YAP1 expression *in vivo*, the AAV9 vector pAAV9-GfaABC1D-YAP1-3×Flag-WPRE (AAV-YAP1, purchased from Obio Technology) was employed, while pAAV9-GfaABC1D-MCS-WPRE (AAV-Con) served as the control. Similarly, to upregulate exogenous NEDD4 expression *in vivo*, the AAV9 vector pAAV9-GfaABC1D-NEDD4 -3×Flag-WPRE (AAV- NEDD4, purchased from Obio Technology) was employed. Immediately after inducing SCI, the mice were positioned in a stereotaxic frame, and viral injections were carried out at four peripheral sites surrounding the lesion. At each site, 1 μl of viral solution containing 5 × 10⁹ vector genomes (vg) were delivered at a rate of 200 nl/min to a depth of 1 mm. Injections were administered with a 10-μl Neuros™ syringe (Hamilton Company, Reno, NV, USA) and a precision microinjection pump (RWD Life Science, Shenzhen, China).

### Reverse transcription-quantitative PCR (RT-qPCR)

Using TRIzol reagent (Invitrogen), total RNA from spinal cord tissues and primary astrocytes was isolated, followed by cDNA synthesis from 1 μg RNA with the PrimeScript RT Kit (Takara, Japan). RT-qPCR was conducted on a real-time PCR system using the TB Green® Premix Ex Taq™ kit (Takara, Japan). Gene expression levels were normalized to *Actb* (β-actin) and calculated using the 2^^-ΔΔCT^ method. The primer sequences used were as follows: *Yap1* primer: forward 5'-CCAGACGACTTCCTCAACAGTG-3' and reverse 5'-GCATCTCCTTCCAGTGTGCCAA-3'. *Nedd4* primers: forward 5'-GAGTGGAATCCTTACCAGCGTG-3' and reverse 5'-AGAATGCGGTGTCGCTGTGGAA-3'. *Foxm1* primer: forward 5'-GTCTCCTTCTGGACCATTCACC-3' and reverse 5'-GCTCAGGATTGGGTCGTTTCTG-3'. *Actb* primer: forward 5'-GAGCTGCGTTTTACACCCT-3' and reverse 5'-GCCTT CACCGTTCCAGTTTT-3'.

### Western blot analysis

Protein lysates were prepared from spinal cord tissue (a 4 mm segment centered on the lesion site) and cultured cells using a lysis buffer (Beyotime, China) according to the manufacturer's instructions. Equal amounts of protein were separated by SDS-PAGE, transferred to PVDF membranes, and blocked with 5% BSA at room temperature. After overnight incubation at 4 °C with primary antibodies, membranes were incubated for 2 hours with HRP-conjugated secondary antibodies (Jackson ImmunoResearch, USA). Protein bands were visualized using enhanced chemiluminescence (ECL) and quantified with ImageJ software. The primary antibodies used included: Mouse anti-YAP1 (sc-101199, Santa Cruz); Rabbit anti-YAP1 (phospho S127, EP1675Y, Abcam), Mouse anti-NEDD4 (sc-518160, Santa Cruz); Rabbit anti-Ubiquitin (Ub; 10201-2-AP, Proteintech); Mouse anti-β-Actin (66009-1-Ig, Proteintech); Rabbit anti-FOXM1 (13147-1-AP, Proteintech); Rabbit anti-ATG5 (10181-2-AP, Proteintech); Rabbit anti-BECN1 (11306-1-AP, Proteintech); Rabbit anti-GFAP (GB11096, Servicebio); Rabbit anti-Flag (80010-1-RR, Proteintech); Mouse anti-HA (66006-2-Ig, Proteintech); Mouse anti-His (66005-1-Ig, Proteintech); Mouse anti-Myc (60003-2-Ig, Proteintech).

### Immunoprecipitation

Astrocytes and HEK293T cells were harvested and lysed as previously described. The co-immunoprecipitation (Co-IP) assay was carried out using the Pierce™ Crosslink Magnetic IP/Co-IP Kit (88805, Thermo Fisher Scientific), where cell lysates were incubated with magnetic beads pre-bound to specific antibodies, as per the instructions provided by the manufacture. The immune complexes were washed five times with lysis buffer to remove non-specifically bound proteins, and the precipitated proteins were subsequently analyzed by Western blotting.

For silver staining and immunoprecipitation followed by mass spectrometry (IP-MS), protein lysates were obtained from primary astrocytes transfected with either Flag-YAP1 or Flag-Vector constructs. These extracts were incubated with anti-Flag magnetic beads to isolate YAP1-associated protein complexes. Bound proteins were eluted by boiling and resolved via SDS-PAGE. The resulting gels were subjected to silver staining (Beyotime, China) or analyzed by mass spectrometry (Thermo Fisher Scientific, USA) according to the respective manufacturers' instructions. For MS, protein bands were excised from the gel and subjected to in-gel digestion with trypsin. Peptides were then extracted and analyzed using a mass spectrometer. Data was acquired in a data-dependent acquisition mode, and the resulting spectra were processed and searched against the UniProt database using the MaxQuant software. Peptide identification and quantification were carried out using standard settings, with a false discovery rate (FDR) of less than 1% for both peptide and protein identification.

### Statistical analysis

Data are expressed as mean ± standard error of the mean (SEM), with statistical analysis carried out using GraphPad Prism (version 8.0, GraphPad Software Inc., USA). Independent samples Student's t-test was used to compare two groups, while one-way or two-way analysis of variance (ANOVA) with *post hoc* Bonferroni correction was applied for comparisons among more than two groups. A p-value of <0.05 (two-tailed) was deemed statistically significant for all analyses. (*P < 0.05, **P < 0.01, ***P < 0.001).

## Results

### YAP1 was upregulated in astrocyte after SCI

**Figure 1:**
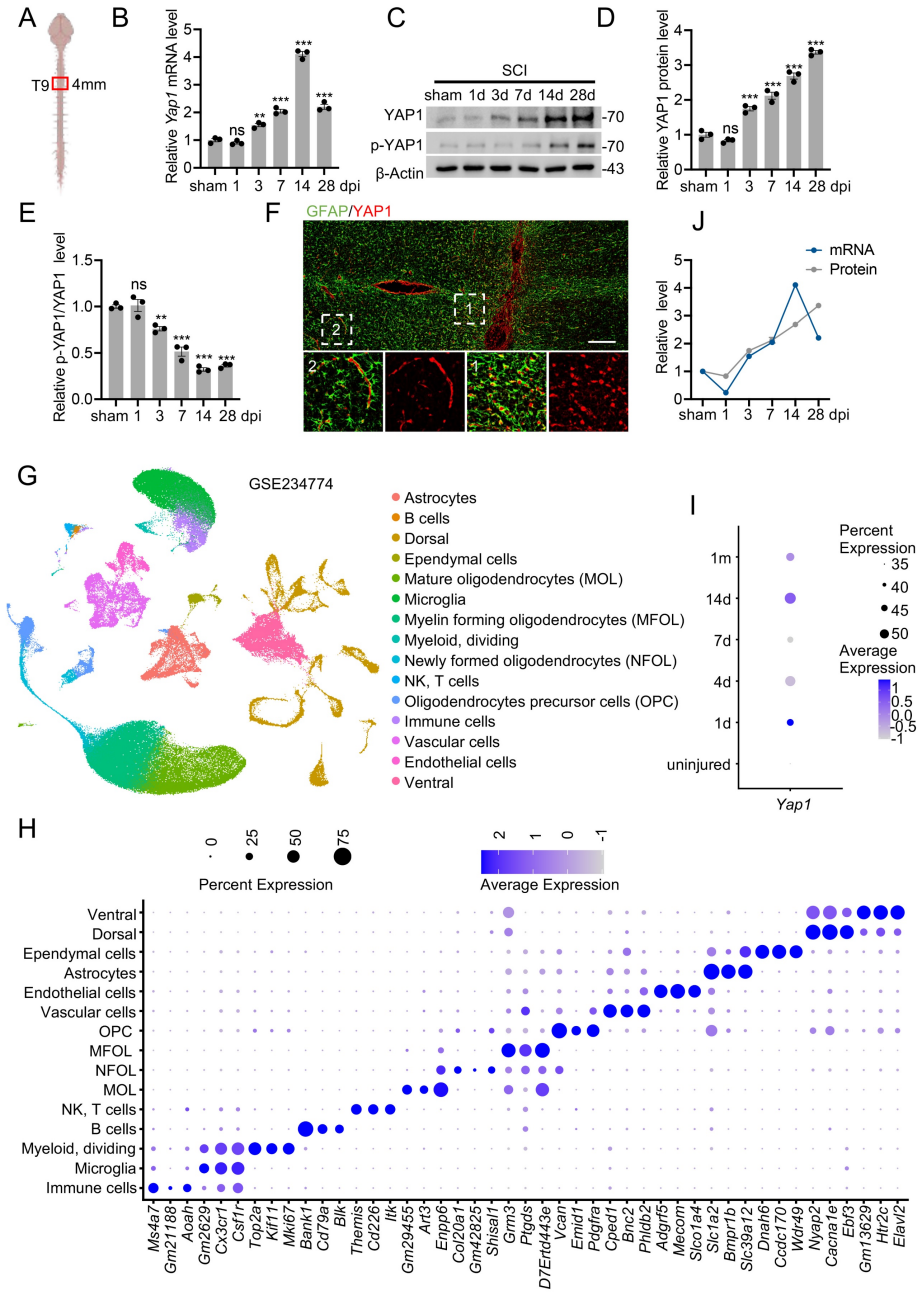
** YAP1 was upregulated in astrocyte after SCI.** (A) Schematic representation of the site of mouse injured spinal cord tissue used for WB and RT-qPCR. (B) Temporal changes in *Yap1* mRNA expression in the mouse spinal cord following SCI (n = 3). (C-E) Protein expression and quantification of YAP1 and p-YAP1 of injured spinal cord at the indicated time after SCI (n = 3). (F) Double immunofluorescence staining of YAP1 (red) and GFAP (green); Images of selected regions (white squares) are shown at higher magnification (Scare bar = 200μm). (G) Uniform Manifold Approximation and Projection (UMAP) plot illustrating distinct clusters of spinal cord cells following SCI (GSE234774). (H) Dot plot displaying marker genes used for cell-type annotation in (G). (I) Dot plot showing the *Yap1* expression in astrocytes at the indicated times. (J) Temporal expression of YAP1 mRNA and protein expression levels in injured spinal cords at different time points. Statistical analysis for panels B, D and E was performed using one-way ANOVA with Bonferroni's *post hoc* correction.

To investigate YAP1 expression following SCI, we first quantified its mRNA and protein levels at the lesion site using RT-qPCR and Western blotting, respectively (Fig. 1A). Both YAP1 mRNA and protein levels were significantly elevated in SCI mice compared to the sham group (Fig. 1B-D). In addition, the ratio of p-YAP1 to total YAP1 gradually decreased following SCI (Fig. 1C, E). To identify the specific cell types expressing YAP1 post-injury, we performed co-immunostaining of YAP1 with astrocyte, neuronal, and macrophage/microglial markers. Immunofluorescence analysis demonstrated that YAP1 expression was predominantly localized to astrocytes (Fig. 1F; Fig. S1A, B). Interestingly, YAP1 was primarily cytoplasmic in astrocytes distant from the lesion, while in reactive astrocytes near the lesion epicenter, it was mainly nuclear. Furthermore, YAP1 protein expression was markedly increased in primary astrocytes subjected to *in vitro* scratch injury (Fig. S1C, D). Single-cell RNA sequencing (scRNA-seq) data from SCI mice confirmed an upregulation of *Yap1*, peaking at 14 days post-injury (dpi), consistent with our findings (Fig. 1G-I; Fig. S1E-G). Notably, although YAP1 mRNA levels declined after 14 dpi, protein levels remained persistently high (Fig. 1J). This discrepancy suggests that post-translational modifications (PTMs) may contribute to the sustained elevation of YAP1 protein following SCI.

### NEDD4 interacts with YAP1

**Figure 2:**
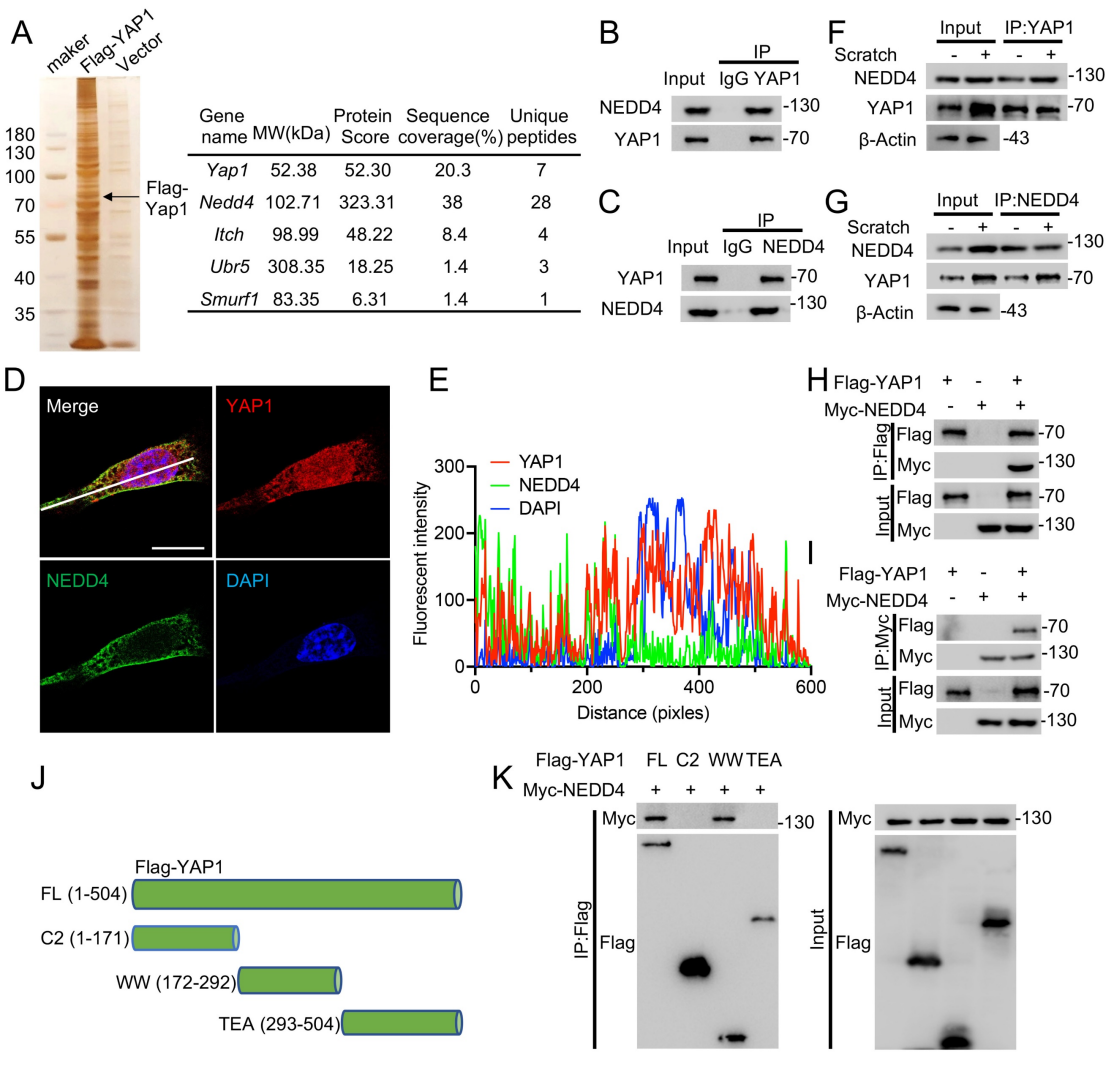
** NEDD4 interacts with YAP1.** (A) Immunoprecipitated gels from primary astrocytes were subjected to silver staining, and MS analysis identified NEDD4 as a YAP1-interacting protein. (B-C) Co-IP assays were conducted to investigate the interaction between endogenous NEDD4 and YAP1 in primary astrocytes. (D-E) Primary astrocytes were immunofluorescence stained using anti-NEDD4 and anti-YAP1 (D) (Scare bar = 50μm). Pearson's correlation analysis was applied for quantitative assessment (E). (F-G) Following scratch injury or control treatment, primary astrocytes were collected after 48 h, and protein lysates were subjected to immunoprecipitation with anti-YAP1 (F) and anti-NEDD4 (G), followed by immunoblotting with the indicated antibodies. (H-I) Co-IP assays were carried out to investigate the interaction between exogenous NEDD4 and YAP1 in HEK293T cells. (J) Schematic diagram showing Flag-tagged full-length and truncated forms of YAP1. (K) HEK293T cells were co-transfected with Myc-NEDD4 and Flag-YAP1 or mutant plasmids. Lysates were immunoprecipitated with anti-Flag and immunoblotted with the indicated antibodies.

To identify potential YAP1-interacting proteins, Co-IP was performed using lysates from primary astrocytes expressing Flag-tagged YAP1. Protein complexes were pulled down using anti-Flag beads and subsequently analyzed by MS (Fig. 2A). Given the regulatory role of E3 ubiquitin ligases in post-translational modification, particular attention was paid to this class of proteins. Four E3 ligases—NEDD4, ITCH, UBR5, and SMURF1—were identified in the MS results (Fig. 2A, Fig. S2A). In addition, UbiBrowser prediction analysis identified 13 candidate E3 ligases potentially targeting YAP1 (Fig. S2B), though these interactions were not experimentally confirmed 32. Among the tested candidates, only knockdown of NEDD4 led to a significant reduction in YAP1 protein levels (Fig. S2C). Consistently, NEDD4 expression was found to be markedly upregulated both in the spinal cord post-SCI (Fig. S2D, E) and in primary astrocytes following scratch injury (Fig. S2F, G). To confirm the interaction between endogenous YAP1 and NEDD4, Co-IP was performed on protein lysates from primary astrocytes using anti-YAP1 or anti-NEDD4 antibodies, followed by immunoblotting with the corresponding antibodies (Fig. 2B, C). Immunofluorescence staining further demonstrated cytoplasmic co-localization of YAP1 and NEDD4 (Fig. 2D, E). Upon scratch injury, an increased amount of YAP1 was co-immunoprecipitated with NEDD4, and a similar enhancement of the YAP1-NEDD4 interaction was observed in astrocytes subjected to stretch injury (Fig. 2F, G). To validate this interaction in a heterologous system, HEK293T cells were co-transfected with Flag-YAP1 and Myc-NEDD4 constructs. Co-IP assays confirmed that Myc-NEDD4 efficiently co-precipitated with Flag-YAP1 (Fig. 2H, I), supporting interaction between the two proteins. To map the interaction domain, a series of Flag-YAP1 deletion mutants were generated (Fig. 2J) and tested in co-transfected HEK293T cells. The Co-IP results revealed that the WW domain (amino acids 172-292) of YAP1 is essential for its binding to NEDD4 (Fig. 2K).

### NEDD4 stabilizes YAP1 through the inhibiting CMA pathway degradation

**Figure 3:**
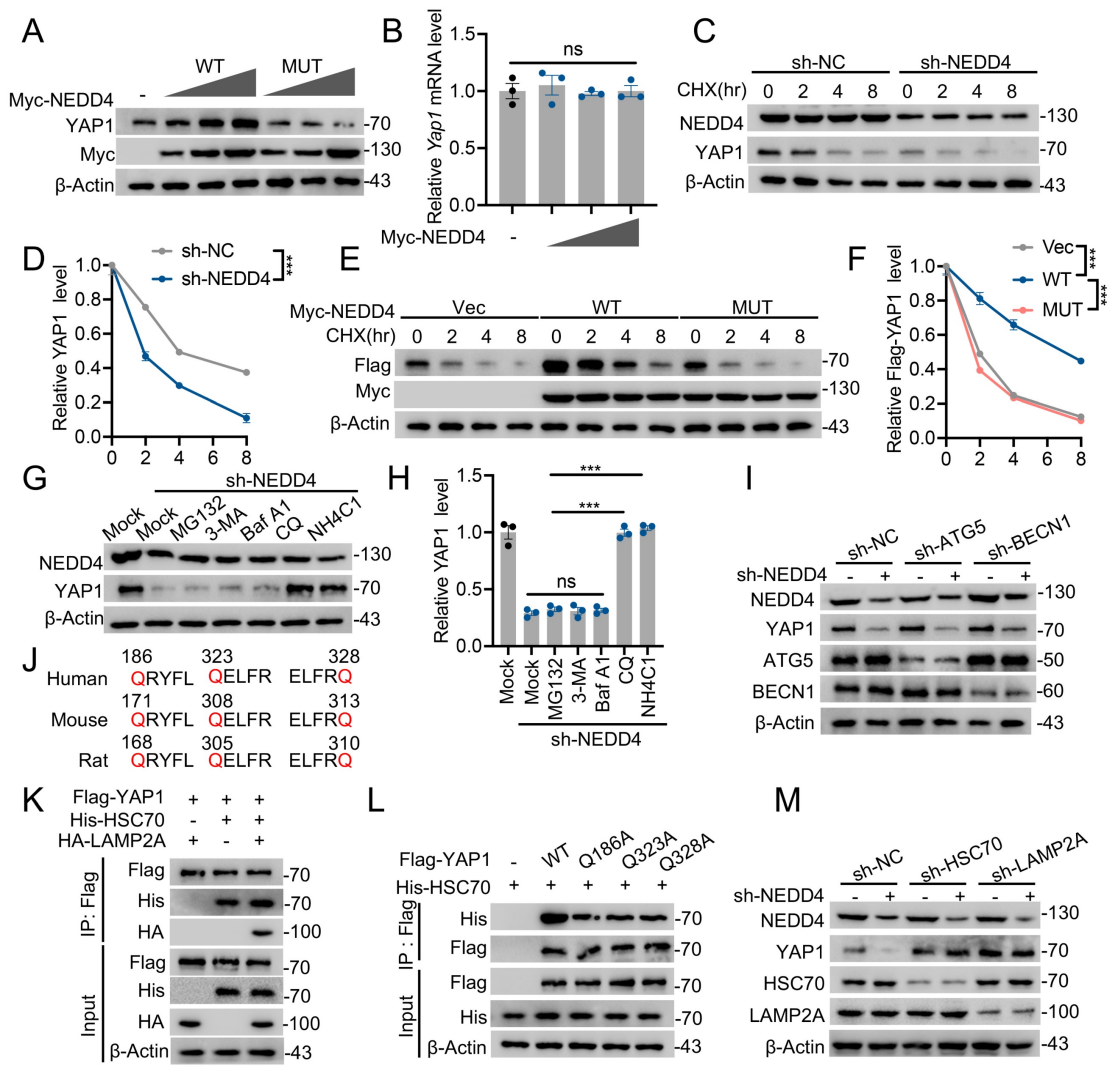
** NEDD4 stabilizes YAP1 through the inhibiting CMA pathway degradation.** (A) Increasing amounts of Myc-tagged NEDD4 (WT or MUT) were transfected into primary astrocytes and cell lysates were analyzed using immunoblotting with anti-YAP1 and anti-Myc. (B) *YAP1* mRNA levels in primary astrocytes transfected with control and different amounts of Myc-NEDD4. (C-D) YAP1 protein expression in shNC- and shNEDD4-transfected astrocytes was analyzed at the indicated time points after treatment with CHX (10 μg/ml) (n = 3). (E-F) Indicated protein levels in HEK293T transfected Flag-YAP1with or without Myc-tagged NEDD4 (WT or MUT) using CHX (10 μg/ml) and quantified (n = 3). (G-H) YAP1 protein levels in primary astrocytes transfected with shNEDD4 and treated with DMSO (Mock), MG132, 3-MA, Baf A1, CQ, or NH_4_CI and quantification (I) Primary astrocytes transfected with scramble shRNA or ATG5- or BECN1-specific shRNAs were co-transfected with shNC or shNEDD4 and lysates were analyzed using immunoblotting. (J) The presence of three canonical KFERQ-like motifs in YAP1. (K) HEK293T cells were co-transfected with Flag-YAP1, His-HSC70, and HA-LAMP2A. Cell lysates were collected to assess the interaction between Flag-YAP1, His-HSC70, and HA-LAMP2A. (L) HEK293T cells were co-transfected as indicated. Co-IP analysis of His-HSC70 interaction with Flag- YAP1 WT or mutants. (M) Primary astrocytes transfected with scramble shRNA or HSC70- or LAMP2A-specific shRNAs were co-transfected with shNC or shNEDD4 and lysates were analyzed using immunoblotting. Statistical analyses were performed using one-way ANOVA for panel H and two-way ANOVA for panels D and F, followed by Bonferroni's *post hoc* correction.

Since NEDD4 knockdown inhibits YAP1 protein expression in astrocytes, the biochemical mechanism by which NEDD4 regulates YAP1 stability in astrocytes was next investigated. Overexpression of WT NEDD4 enhanced YAP1 protein levels, while overexpression of the catalytically inactive NEDD4 MUT did not elicit a similar effect (Fig. 3A), suggesting that the E3 ligase activity of NEDD4 is required for this regulatory process. Notably, RT-qPCR analysis confirmed that NEDD4 overexpression had no detectable impact on *Yap1* transcript levels (Fig. 3B), supporting a post-translational mode of regulation. To determine whether NEDD4 affects the degradation kinetics of YAP1, cycloheximide (CHX) chase assays were performed. In primary astrocytes, NEDD4 depletion markedly accelerated the degradation of endogenous YAP1 (Fig. 3C, D). In HEK293T cells, overexpression of WT NEDD4 stabilized exogenously expressed YAP1 during CHX treatment, whereas the MUT NEDD4 failed to prevent its degradation (Fig. 3E, F). Given that cellular proteostasis is governed by both the ubiquitin-proteasome system (UPS) and lysosome-dependent pathways 33,34, we sought to identify the degradation route responsible for YAP1 turnover in the absence of NEDD4. Pharmacological inhibition experiments revealed that lysosomal inhibitors (NH₄Cl and chloroquine (CQ)) significantly blocked NEDD4 depletion-induced YAP1 degradation, whereas proteasome inhibition (MG132) and blockade of macroautophagy (3-methyladenine (3-MA) and bafilomycin A1 (Baf A1)) exerted minimal effects (Fig. 3G, H). Furthermore, NEDD4 knockdown continued to promote YAP1 degradation in BECN1- or ATG5-deficient cells lacking canonical macroautophagy, indicating that this process is independent of both the UPS and macroautophagy pathways (Fig. 3I). Based on these findings, we hypothesized that YAP1 may be degraded via CMA, a selective lysosomal degradation pathway. In silico analysis identified three KFERQ-like motifs in the YAP1 amino acid sequence, characteristic of CMA-targeting signals (Fig. 3J). Co-IP assays revealed interactions between YAP1 and the CMA components HSC70 and LAMP2A (Fig. 3K). Furthermore, mutation of a single KFERQ-like motif disrupted the YAP1-HSC70 interaction (Fig 3L). Furthermore, knockdown of HSC70 or LAMP2A reversed the YAP1 degradation induced by NEDD4 knockdown (Fig 3M), functionally validating YAP1 as a CMA substrate. Taken together, these findings demonstrate that NEDD4 stabilizes YAP1 at the post-translational level by preventing its degradation through the CMA pathway. Loss of NEDD4 facilitates the recognition and lysosomal targeting of YAP1 via CMA, independent of the proteasome and macroautophagic degradation systems.

### NEDD4 promoted K63-linked ubiquitination of YAP1 at K254

**Figure 4:**
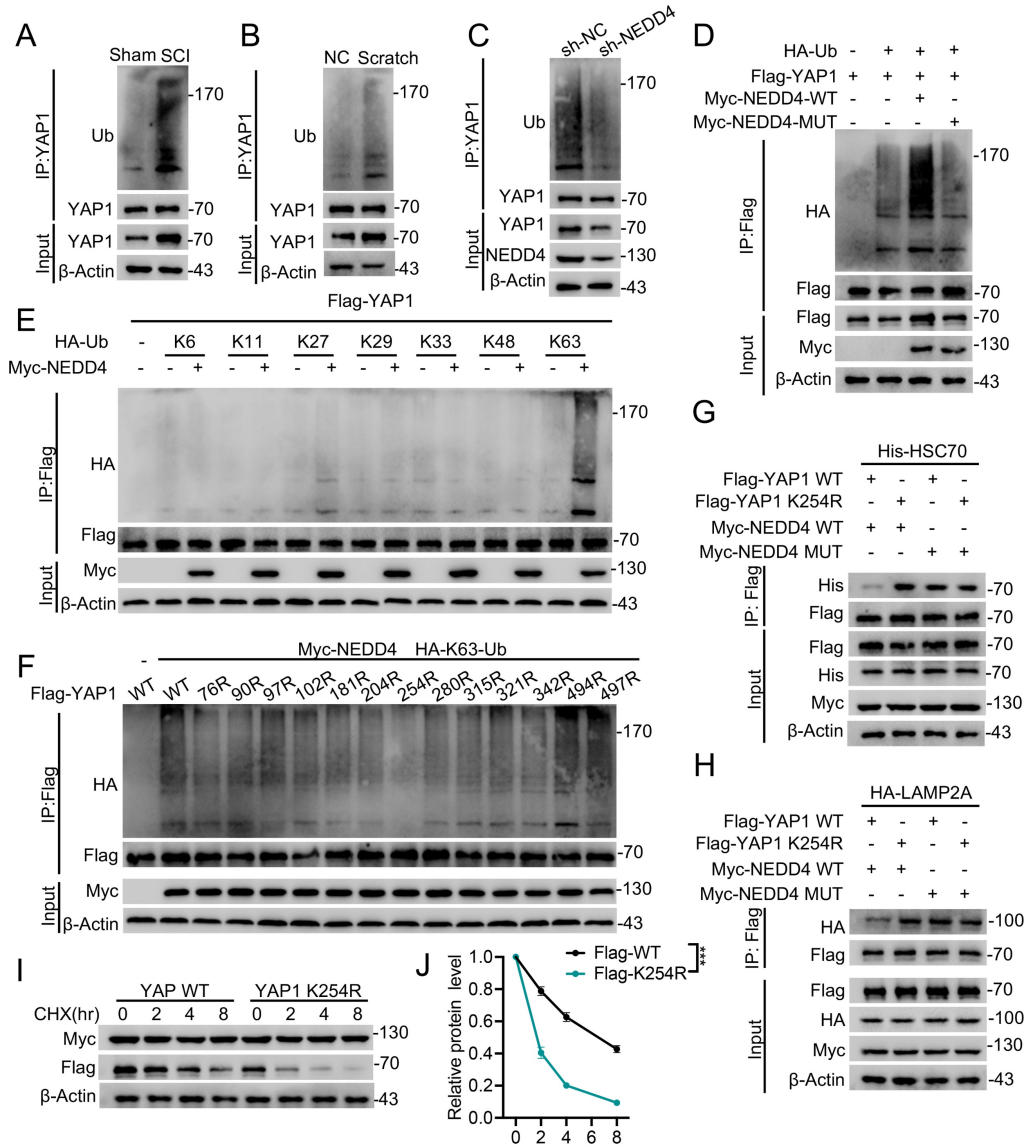
** NEDD4 promoted K63-linked ubiquitination of YAP1 at K254.** (A) I Spinal cord lysates from sham and SCI mice were immunoprecipitated with anti-YAP1 antibody, and ubiquitination was detected using anti-Ub. (B) Lysates from astrocytes, either untreated or subjected to scratch injury, were immunoprecipitated with anti-YAP1 antibody, and ubiquitination was analyzed using anti-Ub. (C) Lysates from primary astrocytes transfected with shNC or shNEDD4 were subjected to immunoprecipitation using anti-YAP1 antibody and analyzed by immunoblotting with the indicated antibodies. (D) HEK293T cells were transfected with HA-Ub, Flag-YAP1, and Myc-NEDD4 (WT or MUT), followed by immunoprecipitation with anti-Flag and immunoblotting with the indicated antibodies. (E) HEK293T cells were co-transfected with Flag-YAP1, Myc-NEDD4, and the indicated HA-Ub plasmids and Flag-YAP1 ubiquitylation linkage was examined. (F) HEK293T cells were co-transfected with Myc-NEDD4, HA-K63 ub and Flag-YAP1 (WT and indicated MUTs) and Flag-YAP1 ubiquitylation was examined. (G) HEK293T cells were co-transfected as indicated. Co-IP analysis of Flag-YAP1 (WT or K254R) interaction with His-HSC70. (H) HEK293T cells were co-transfected as indicated. Co-IP analysis of Flag-YAP1 (WT or K254R) interaction with HA-LAMP2A. (I-J) Lysates from HEK293T cells co-transfected with Flag-YAP1 (WT or K254R) with Myc-NEDD4 were immunoblotted with anti-Flag and anti-Myc in the absence or presence of CHX (10 μg/mL) for indicated time periods and quantified (n = 3). Statistical analysis for panel J was performed using two-way ANOVA with Bonferroni's *post hoc* correction.

Since NEDD4 is an E3 ubiquitin ligase and ubiquitination is closely associated with protein modification 35, we further investigated whether NEDD4 regulates YAP1 CMA degradation via modulating its ubiquitination. First, we examined YAP1 ubiquitination levels in primary astrocytes isolated from injured spinal cords. YAP1 ubiquitination was significantly increased following SCI (Fig. 4A) and similar elevations were observed in astrocytes under *in vitro* injury-mimicking conditions (Fig. 4B). Importantly, knockdown of NEDD4 in primary astrocytes reduced YAP1 ubiquitination (Fig. 4C), whereas overexpression of WT NEDD4 enhanced YAP1 ubiquitination levels. In contrast, the catalytically inactive MUT NEDD4 failed to alter exogenous YAP1 ubiquitination levels in HEK293T cells (Fig. 4D). To further identify the specific type of ubiquitin linkage responsible for NEDD4-mediated YAP1 ubiquitination, we co-transfected HEK293T cells with NEDD4, YAP1, and plasmids encoding various ubiquitin chains, including K6-, K11-, K27-, K29-, K33-, K48-, and K63-linked forms. Among these, NEDD4 selectively and robustly enhanced K63-linked ubiquitination of YAP1, suggesting that K63-linked polyubiquitination mediates NEDD4-dependent YAP1 stabilization (Fig. 4E). To pinpoint the exact ubiquitination site on YAP1 responsible for this modification, we generated 13 YAP1 lysine-to- arginine (K→R) mutants, each with a single lysine substitution. Co-transfection of these mutants into HEK293T cells with Flag-YAP1 and His-tagged K63-ubiquitin revealed that only the K254R significantly abolished YAP1 K63-linked ubiquitination (Fig. 4F). Notably, the K254R also enhanced the interaction between YAP1 and HSC70 (Fig. 4G). Similarly, the K254R also enhanced the interaction between YAP1 and LAMP2A (Fig. 4H). Furthermore, in the presence of CHX, NEDD4 was unable to promote the stability of the YAP1-K254R, indicating that ubiquitination at K254 is essential for NEDD4-mediated stabilization and protection of YAP1 from CMA-dependent degradation (Fig. 4I, J).

### Conditional knockout of *Nedd4* in astrocytes inhibited astrogliosis and hindered functional recovery after SCI

**Figure 5:**
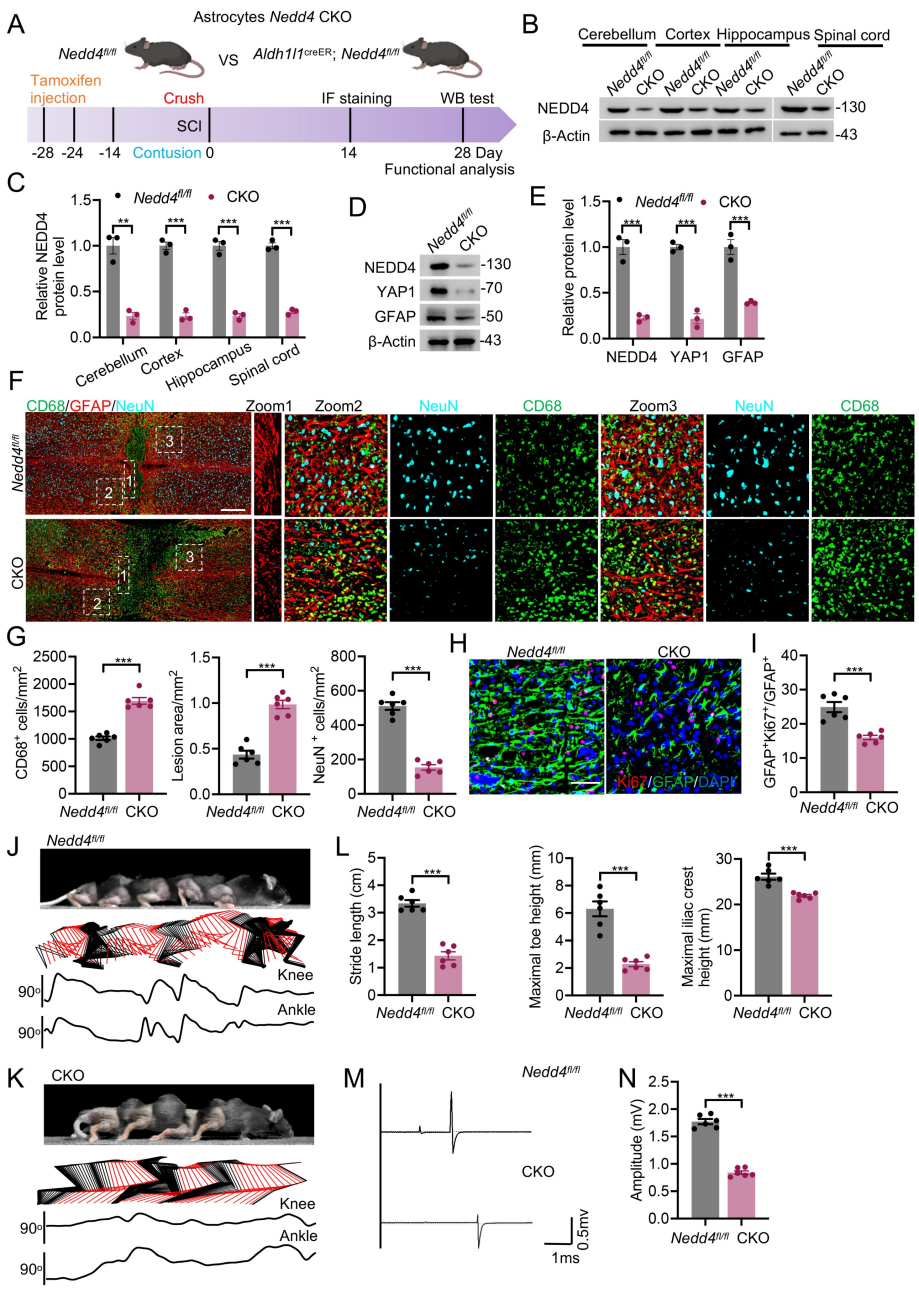
** Conditional knockout of *Nedd4* in astrocytes inhibited astrogliosis and hindered functional recovery after SCI.** (A) Schematic workflow. (B-C) Validation of NEDD4 CKO efficiency using anti-NEDD4 in the mouse cerebellum, cerebral cortex, hippocampus, and spinal cord and quantification. (D-E) Indicated protein levels in primary astrocytes isolated from *Nedd4^fl/fl^* and CKO mice spinal cord and quantification (n = 3). (F-G) Representative confocal images showing co-staining of CD68, GFAP, and NeuN in spinal cord sections from the indicated groups at 14 dpi, together with quantification of CD68⁺ macrophages/microglia, lesion area, and neuronal number (n = 6). (Scale bar = 200μm). (H-I) Representative confocal images of Ki67 and GFAP co-staining in spinal cord sections from the indicated groups at 14 dpi, accompanied by quantification (n = 6) (Scale bar = 50μm). (J-K) Chronophotographs of *Nedd4^fl/fl^* and CKO SCI mice at 28 dpi together with color-coded stick-figure representations of hindlimb movements, plotted joint angle excursions of the knee and ankle. (L) Measurements of stride length, maximal iliac crest height and maximal toe elevation under indicated condition. (n = 6). (M-N) Representative EMG recordings of the gastrocnemius muscle, and their quantification in mice at 28 dpi (n = 6). Statistical analysis for panels C, E, G, I, L and N was performed using two-tailed unpaired Student's t-test.

To investigate the physiological role of astrocytic *Nedd4* following SCI, we established a conditional knockout (CKO) mouse model by crossing *Nedd4^fl/fl^* mice with *Aldh1l1^CreER^* transgenic mice (Fig. 5A). Efficient and specific recombination in astrocytes was achieved following tamoxifen administration, as confirmed by immunoblotting analyses (Fig. 5B, C). In line with previous results, astrocyte-specific ablation of *Nedd4* led to a significant downregulation of YAP1 protein levels (Fig. 5D, E). To delineate the consequences of *Nedd4* loss on astrocytic responses after SCI, we performed a list of analyses comparing *Nedd4^fl/fl^* and CKO mice. Western blotting results demonstrated a notable reduction in astrocytic proliferation in the CKO group, as evidenced by decreased levels of GFAP (Fig. 5D, E). Histopathological assessments revealed that deletion of *Nedd4* in astrocytes significantly worsened lesion pathology, characterized by an expansion of the lesion core, increased infiltration of CD68^+^ macrophages/microglia, and exacerbated neuronal loss relative to control mice (Fig. 5F, G). Consistently, immunofluorescence analysis revealed a significant decrease in the number of Ki67^+^ astrocytes in the CKO group relative to the *Nedd4^fl/fl^* mice, indicating that loss of *Nedd4* compromises the intrinsic proliferative capacity of reactive astrocytes *in vivo* (Fig. 5H, I). In support of this finding, *in vitro* scratch wound assays further demonstrated that knockdown of NEDD4 markedly suppressed astrocyte proliferation (Fig. S3A, B). These histological and cellular alterations were accompanied by marked deficits in functional recovery. *Nedd4^fl/fl^* mice greater angular displacement of hindlimb joints. In contrast, CKO mice exhibited sustained hindlimb dragging and limited weight-bearing capacity throughout the observation period (Fig. 5J-L). Electrophysiological recordings further revealed significantly diminished MEPs in the gastrocnemius muscles of CKO mice, reflecting impaired integrity and conduction of descending motor pathways (Fig. 5M, N). Collectively, these findings indicate that astrocytic *Nedd4* is essential for modulating glial proliferation, maintaining lesion containment, and promoting functional recovery following SCI.

### Overexpression of YAP1 improved functional recovering after SCI

**Figure 6:**
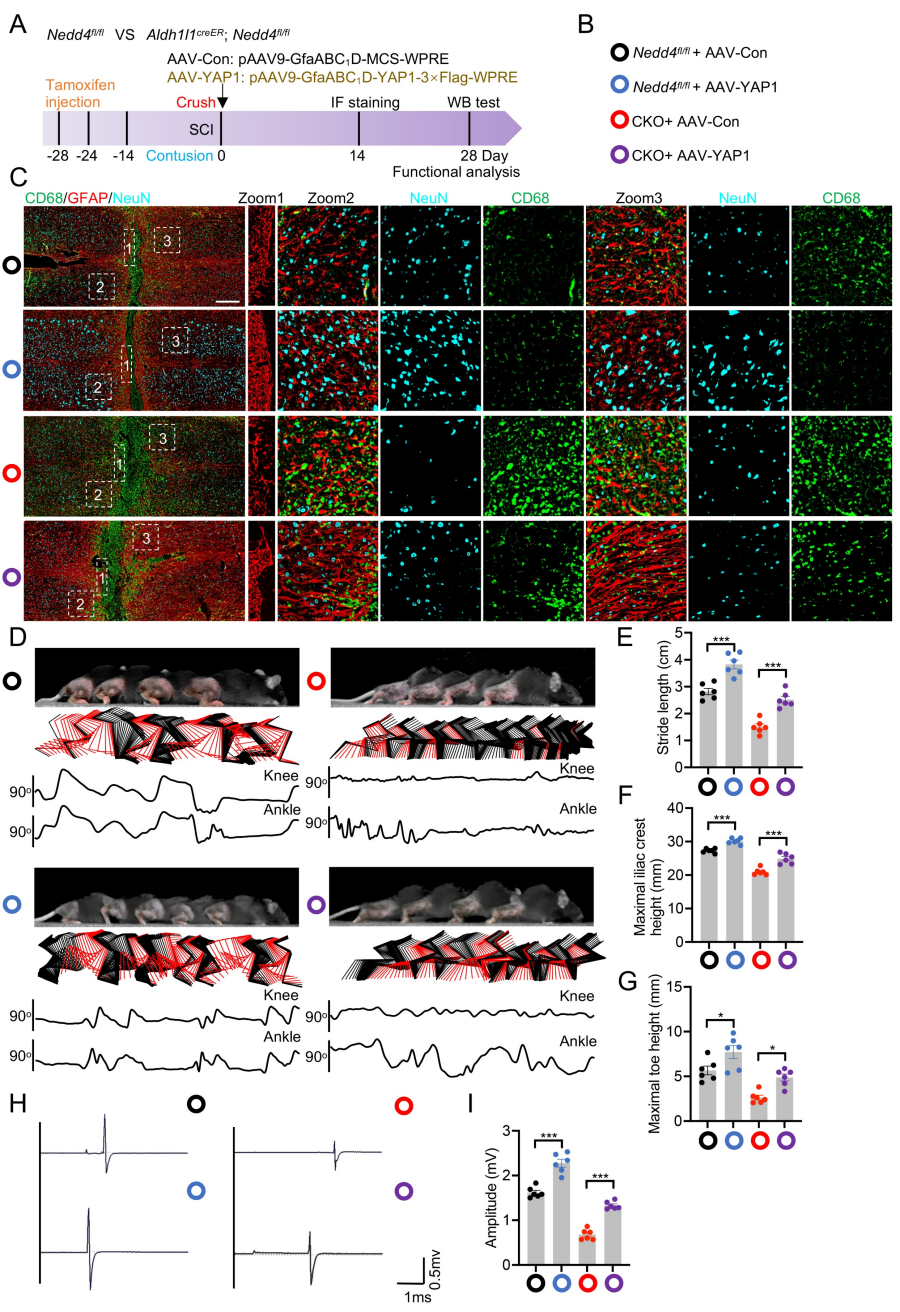
** Overexpression of YAP1 improved functional recovering after SCI.** (A) Schematic workflow. (B) Specific grouping information is indicated by the specified color. (C) Representative confocal images showing co-staining of CD68, GFAP, and NeuN in spinal cord sections from the indicated groups at 14 dpi (n = 6). (Scale bar = 200μm). (D) Chronophotographs of indicated groups at 28 dpi together with color-coded stick-figure representations of hindlimb movements, plotted joint angle excursions of the knee and ankle. Measurements of stride length (E), maximal iliac cres height (F) and maximal toe elevation (G) under indicated condition (n = 6). (H-I) Representative EMG recordings of the gastrocnemius muscle, and their quantification in mice at 28 dpi (n = 6). Statistical analysis for panels E, F, G, and I was performed using two-tailed unpaired Student's t-test.

AAV-mediated gene therapy has emerged as a highly sophisticated and increasingly adopted modality for the treatment of SCI, owing to its exceptional biosafety and its ability to elicit sustained, cell type specific transgene expression. Remarkably, AAV vectors possess the capacity to traverse the blood-spinal cord barrier with high efficiency, thereby enabling *in vivo* transduction of genes encoding neuroprotective or regenerative factors directly within the pathological microenvironment of the injured spinal cord. To determine whether NEDD4 regulates functional recovery through selective modulation of YAP1, we administered AAV-YAP1 into both *Nedd4^fl/fl^* and CKO mice to induce astrocytic YAP1 overexpression. Control groups received AAV-Con expressing a non-functional construct (Fig. 6A; Fig. S4A). Experimental groups were visually distinguished by color coding in Fig. 6B. In line with the previously observed NEDD4-dependent astrocyte reactivity, overexpression of YAP1 in the injured spinal cord led to a significant increase in GFAP expression, indicating enhanced astrocyte activation (Fig. S4B-E). Histopathological analysis further revealed that astrocytic YAP1 overexpression ameliorated the aggravated pathology seen in *Nedd4*-deficient mice. Specifically, AAV-YAP1 treatment resulted in a smaller lesion core area, reduced infiltration of CD68^+^ macrophages/microglia, and decreased neuronal loss compared to AAV-Con-treated controls (Fig. 6C). Importantly, these molecular and histological improvements were paralleled by functional recovery. AAV-YAP1-treated CKO mice demonstrated markedly enhanced hindlimb locomotor performance, as evidenced by more coordinated hindlimb movements, consistent dorsally directed stepping patterns, and increased angular displacement of hindlimb joints (Fig. 6D-G). Electrophysiological recordings further supported these findings: MEPs recorded from the gastrocnemius muscle were significantly improved in the AAV-YAP1 group, reflecting better conduction along descending motor pathways (Fig. 6H, I).

### Overexpression of NEDD4 facilitates functional restoration after SCI

To explore the therapeutic efficacy of NEDD4 as a potential target for SCI, mice with SCI were treated with AAV-NEDD4 or AAV-Con. Experimental findings revealed that animals receiving AAV-NEDD4 treatment exhibited a significantly reduced lesion core area, accompanied by decreased infiltration of CD68⁺ macrophages/microglia, enhanced neuronal survival (Fig. S5A-D), and increased reactive astrogliosis (Fig. S5E, F). Furthermore, AAV-mediated NEDD4 overexpression resulted in remarkable functional recovery, as demonstrated by improved hindlimb motor performance (Fig. S5G-K) and restored neural conduction efficiency in electrophysiological assessments (Fig. S5L, M). Collectively, these results highlight the pivotal role of NEDD4 in promoting neuroprotection, glial activation, and tissue regeneration following SCI.

### ROS enhances YAP1 ubiquitination via the FOXM1-NEDD4 axis

**Figure 7:**
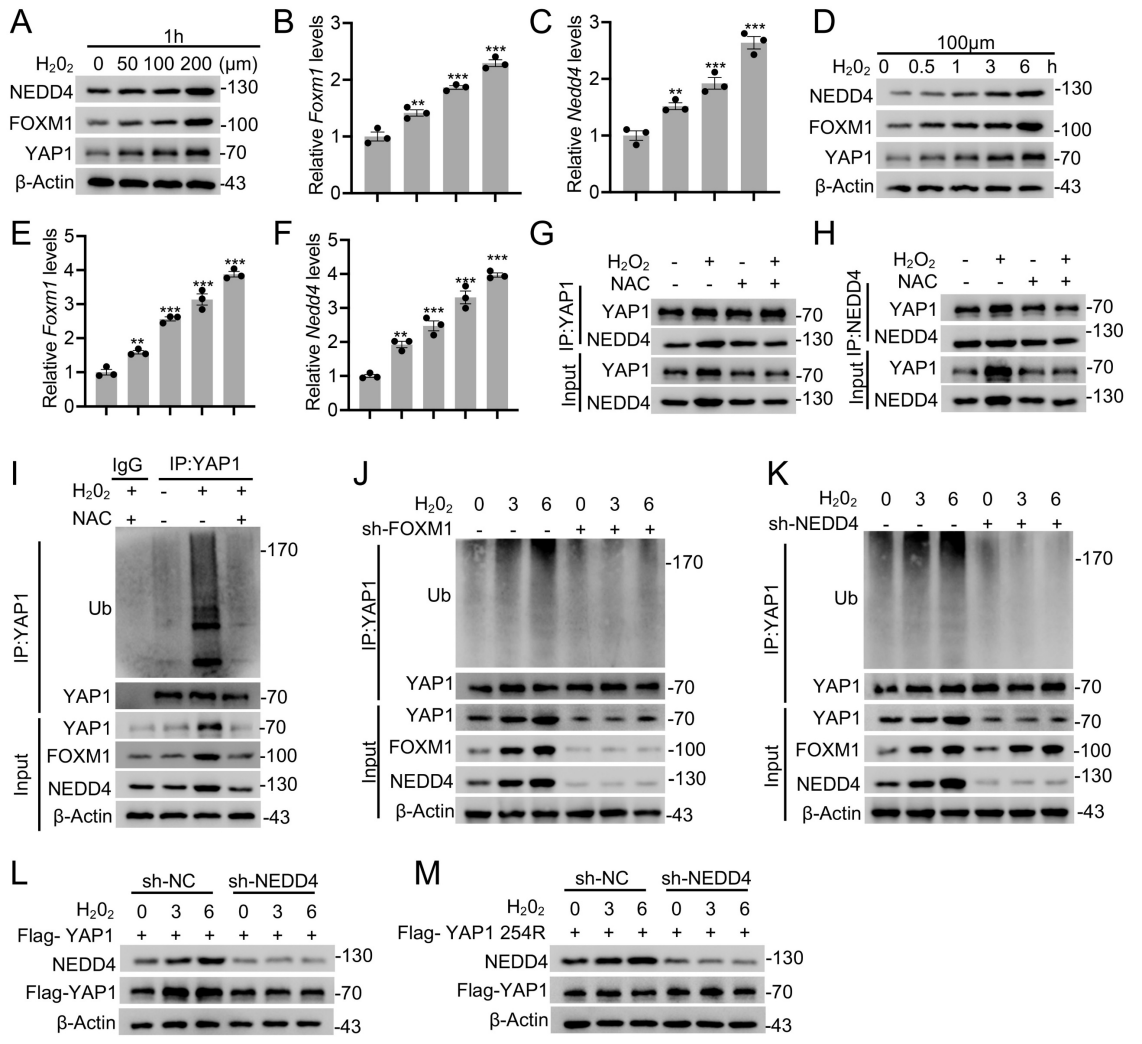
** ROS enhances YAP1 ubiquitination via the FOXM1-NEDD4 axis.** Immunoblotting with anti-NEDD4, anti-FOXM1, and anti-YAP1 antibodies was performed on lysates derived from primary astrocytes exposed to varying H_2_O_2_ concentrations for 1 h (A) or to 0.1 mM H_2_O_2_ for different time intervals (D). Corresponding *Foxm1* and *Nedd4* transcript levels were analyzed by RT-PCR in panels B-C and E-F, respectively. (G-H) Co-IP analysis showing the association between endogenous NEDD4 and YAP1 in primary astrocytes treated with H₂O₂ or NAC. (I) Equal amounts of protein lysates from astrocytes treated with 0.1 mM H₂O₂ for 1 h *in vitro* were immunoprecipitated using an anti-YAP1 antibody and probed with the indicated antibodies. (J-K) Co-IP analysis of lysates from primary astrocytes transfected with sh-FOXM1 (J) or sh-NEDD4 (K) and treated with 0.1 mM H₂O₂ for 0-6 h using anti-YAP1 antibody, followed by immunoblotting with the indicated antibodies. (L) WT or (M) K254R in the absence or presence of sh-NEDD4 were analyzed by immunoblotting with anti-Flag and anti-NEDD4 with 0.1 mM H_2_O_2_ treatment for 0 to 6 h. Statistical analysis for panels B, C, E, and F was performed using one-way ANOVA with Bonferroni's *post hoc* correction.

A hallmark of the SCI microenvironment is elevated ROS 36,37. Interestingly, previous study has revealed a regulatory role for the ROS-FOXM1-NEDD4 signaling cascade in attenuating ferroptosis sensitivity 38,39. Inspired by this, we hypothesized that this pathway might also mediate YAP1 ubiquitination and stability through NEDD4. To test this, we first investigated whether ROS affects endogenous expression of FOXM1 and NEDD4 in astrocytes. Primary astrocytes were treated with varying concentrations of H₂O₂ for 1 hour or with 0.2 mM H₂O₂ across different time points. Both mRNA and protein levels of FOXM1 and NEDD4 were found to increase in a dose- and time-dependent manner following H₂O₂ exposure, suggesting ROS induces the expression of these regulators (Fig.7A-F). Notably, the interaction between NEDD4 and YAP1, which was enhanced by oxidative stress, was abolished upon treatment with the ROS scavenger NAC, indicating a ROS-dependent mechanism (Fig. 7G, H). Furthermore, H₂O₂ treatment upregulated YAP1 ubiquitination, reinforcing the notion that oxidative stress induced NEDD4 expression contributes to increased YAP1 ubiquitination (Fig. 7I). To further confirm the role of the ROS-FOXM1-NEDD4 axis in modulating YAP1 ubiquitination and stability, FOXM1 and NEDD4 were selectively silenced in primary astrocytes. Under these conditions, the H₂O₂-induced increase in YAP1 ubiquitination was abolished, highlighting the necessity of FOXM1 and NEDD4 for this response (Fig. 7J, K). Moreover, silencing NEDD4 prevented the ROS-induced elevation of YAP1 protein levels (Fig. 7L). In contrast, this effect was absent in cells expressing the YAP1 K254R, suggesting that NEDD4-mediated ubiquitination at this lysine residue is essential for YAP1 stabilization under oxidative stress (Fig. 7M). Together, these findings demonstrate that the ROS-FOXM1-NEDD4 axis facilitates the ubiquitination and stabilization of YAP1 in astrocytes.

## Discussion

**Figure 8:**
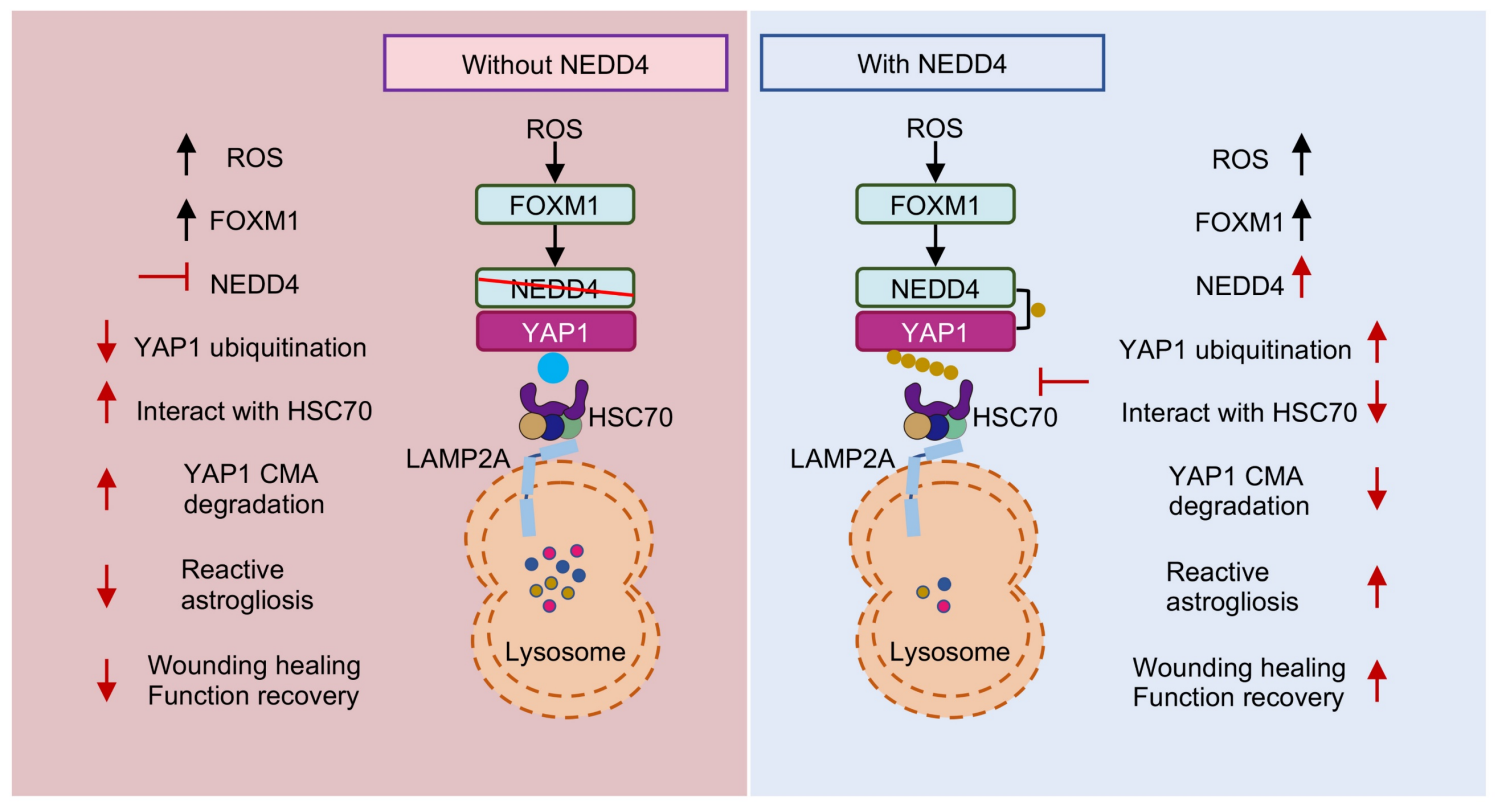
** Proposed mechanism underlying NEDD4 in enhancing reactive astrogliosis after SCI.** NEDD4 promotes K63-linked ubiquitination of YAP1 at K254, thereby preventing the interaction between YAP1 and HSC70 and inhibiting the CMA degradation of YAP1. Activation of the ROS-FOXM1 signaling pathway induces NEDD4 transcription, leading to increased YAP1 stability and subsequent promotion of reactive astrogliosis, wound healing, and functional recovery after SCI. In the absence of NEDD4, YAP1 protein becomes unstable, reactive astrogliosis is suppressed, and locomotor functional recovery after SCI is impaired.

In this study, we identified an unreported functional role for NEDD4 in regulating reactive astrogliosis. NEDD4 catalyzed the conjugation of K63-linked ubiquitin to YAP1 at residue K254, thereby preventing its degradation via HSC70-mediated CMA. This process enhanced YAP1 stability and contributed to the progression of reactive astrogliosis. Following SCI, the ROS-FOXM1 signaling cascade induced NEDD4 expression, further stabilizing YAP1 and promoting astrogliosis. Genetic depletion of *Nedd4* suppressed reactive astrogliosis, exacerbated inflammation, accelerated neuronal loss, and impaired functional recovery post-SCI (Fig. 8).

In response to SCI, astrocytes adjacent to the lesion core are activated to form a dense barrier that limits inflammatory cell infiltration and reduces the injury area 9,10. Additionally, these reactive astrocytes contribute to the repair of the blood-spinal cord barrier and support axon regeneration 40. Nevertheless, the role of reactive astrocytes in SCI remains a subject of ongoing debate. It was once widely believed that the extracellular matrix deposition and the physical barrier generated by astrocytic scar formation constitute major obstacles to neural regeneration 41. However, emerging evidence has challenged this dogma by demonstrating that disruption of glial scar formation exacerbates inflammatory spread, impairs blood-brain barrier repair, increases neuronal tissue loss, and ultimately diminishes functional recovery 9. Furthermore, enhancing astrocytic proliferation has been shown to reduce lesion size and markedly attenuate both inflammation and neuronal injury 42,43. Therefore, a comprehensive investigation of the mechanisms underlying reactive astrogliosis is crucial for improving SCI treatment.

YAP1 translocated from the cytoplasm into the nucleus, where it functions as a transcriptional co-factor by binding to TEAD, thereby regulating cell proliferation, organ growth, and cell differentiation 44,45. Recent studies have revealed that YAP1 is subject to a variety of PTMs, which critically influence its stability, localization, and activity 46,47. Among these, phosphorylation is a key regulatory mechanism. The Hippo signaling pathway phosphorylates YAP/TAZ via the LATS1/2 kinase cascade, promoting their interaction with 14-3-3 proteins and subsequently facilitating their ubiquitination and proteasomal degradation 14,48. Additionally, MAP3K3 has been shown to phosphorylate YAP at Ser405, preventing FBXW7 binding and reducing lysosome-mediated degradation 49. Ubiquitination also plays a central role in controlling YAP1 levels. Several E3 ubiquitin ligases promote YAP degradation. For instance, RNF31 mediates polyubiquitination of YAP at Lys76 in triple-negative breast cancer (TNBC) cells, thereby suppressing tumor growth, migration, and metastasis 50. Similarly, RBCK1 facilitates K48-linked polyubiquitination at Lys76, Lys204, and Lys321, leading to YAP degradation and enhanced apoptosis, which inhibits TNBC progression 51. In contrast, certain factors stabilize YAP1. TRIM11 enhances YAP stability by promoting monoubiquitylation and blocking K11/K48-linked polyubiquitination 52. Likewise, TRIM19 (PML) mediates SUMOylation of YAP at Lys97 and Lys242, thereby preventing its ubiquitination and degradation 53. SETD7-mediated methylation of YAP similarly impairs its ubiquitination and contributes to its stabilization 54.

While the regulation of YAP1 turnover via the UPS has been extensively characterized, the role of CMA in maintaining YAP1 homeostasis remains largely unclear. CMA is a selective lysosomal degradation pathway that targets proteins containing a KFERQ-like motif 55,56. These substrates are recognized by the HSC70 chaperone complex, delivered to the lysosomal membrane receptor LAMP-2A, and translocated into the lysosomal lumen for degradation 57. Previous study has identified YAP1 as a CMA substrate implicating CMA in regulating YAP1 turnover 58. PTMs outside the canonical CMA motif can induce conformational changes that either expose or mask the motif, thereby influencing the CMA targeting of proteins. For instance, acetylation of MST1 at a site distant from the canonical CMA motif inhibits its lysosomal degradation, with HSC70 binding to the canonical motif only after deacetylation 59. In addition to acetylation, emerging evidence suggests that substrate ubiquitination plays a pivotal role in regulating CMA. For example, linear ubiquitination of HIF1α at Lys362 by LUBAC impairs its interaction with HSC70 and LAMP2A, thereby blocking CMA-mediated degradation 60. Moreover, STUB1-mediated K63-linked ubiquitination is essential for the CMA-dependent degradation of HIF1α 61. Similarly, our study shows that NEDD4 catalyzes K63-linked ubiquitination of YAP1 at Lys254, which prevents its recognition by HSC70 and inhibits CMA-mediated degradation, resulting in YAP1 stabilization during reactive astrogliosis. These findings uncover a novel regulatory axis between ubiquitination and CMA in the control of YAP1 turnover. Given the emerging complexity of post-translational regulation, it will be important for future studies to explore whether CMA-mediated degradation of YAP1 is influenced by other modifications, such as SUMOylation, methylation, and palmitoylation.

NEDD4, an E3 ubiquitin ligase, plays a critical role in regulating protein stability through various mechanisms, as evidenced by recent studies. NEDD4 facilitates the ubiquitination of DMT1, promoting its degradation and alleviating ferroptosis 62. NEDD4 catalyzes K27-linked polyubiquitination of TBK1, leading to its recognition by the NDP52 receptor and subsequent selective autophagic degradation 63. Our study reveals that NEDD4 catalyzes K63-linked ubiquitination of YAP1, preventing its degradation through CMA and stabilizing it during reactive astrogliosis after SCI. Although minor ubiquitination signals were detected at K76 and K342, these residues may undergo occasional modification due to local structural accessibility or ubiquitin chain propagation, rather than representing primary catalytic targets. This suggests that while multiple lysine residues on YAP1 could be transiently modified, Lys254 serves as the major functional site mediating K63-linked ubiquitination and YAP1 stabilization. This finding broadens our understanding of how NEDD4 controls protein stability through different ubiquitin linkages, influencing multiple degradation pathways.

Given the significant role of NEDD4 in various diseases, inhibiting NEDD4 has shown remarkable potential in therapeutic applications 23,64. However, specific agonists targeting NEDD4 remain unavailable. Our study further suggests that ROS is a key mediator in the activation of the FOXM/NEDD4/YAP1 axis and the subsequent proliferation of astrocytes. At the same time, it is well recognized that excessive ROS can trigger multiple forms of cell death, including apoptosis, autophagy, and ferroptosis 65. Thus, maintaining ROS homeostasis is essential for neuronal survival and axonal regeneration. Within this context, the NEDD4-YAP1 axis emerges as a particularly attractive therapeutic target. Future studies should aim to identify NEDD4-specific ligand agonists that could enhance astrocytic reactive proliferation while preserving redox balance, thereby improving functional outcomes after SCI. In addition, virtual drug screening offers a promising approach to discover candidate compounds or small molecules capable of selectively enhancing the interaction between NEDD4 and YAP1 66. Such strategies may ultimately lead to novel therapeutic interventions that harness the beneficial aspects of astrocyte reactivity to promote structural repair and functional recovery following SCI.

In summary, we identified a ROS-FOXM1-NEDD4-YAP1 signaling axis that regulates reactive astrogliosis by suppressing CMA-mediated degradation of YAP1. Our results reveal a previously unrecognized role for NEDD4 in astrocyte activation and suggest that it may act as a regulatory node in YAP1 stability, potentially relevant for therapeutic intervention following SCI.

## Supplementary Material

Supplementary figures.
