# Supplementary material for: NEDD4 promotes reactive astrogliosis by enhancing K63-linked ubiquitination and inhibiting chaperone-mediated autophagy degradation of YAP1
Source: Int J Biol Sci. 2026 Jan 1;22(2):858–75. doi: 10.7150/ijbs.123639 (PMC12781170; doi:10.7150/ijbs.123639)
Supplement: Supplementary file 1 — Supplementary figures. [file ijbsv22p0858s1.pdf]

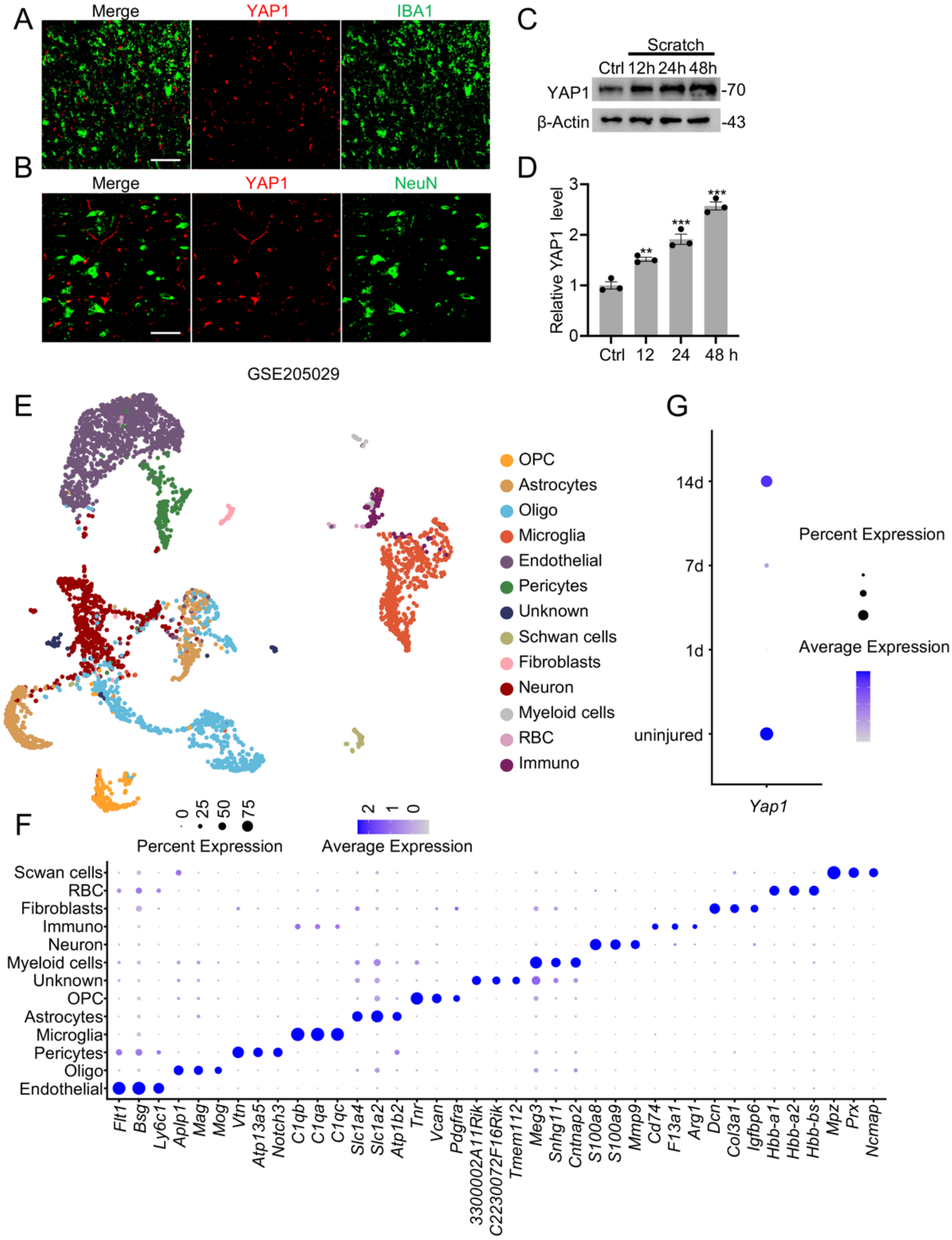

Fig. S1 YAP1 was upregulated after SCI. (A) Double immunofluorescence staining of YAP1 (red) and IBA1 (green) (Scare bar = 50μm). (B) Double immunofluorescence staining of YAP1 (red) and NeuN (green) (Scare bar =50μm). (C-D) Protein expression and quantification of YAP1 of astrocyte at the indicated time after *vitro* scratch injury(n=3). (E) UMAP plot illustrating distinct clusters of spinal cord cells following SCI (GSE205029). (F) Dot plot displaying marker genes used for cell-type annotation in (E). (G) Dot plot showing the *Yap1* expression in astrocytes at the indicated times.

Statistical analysis for panel D was performed using one-way ANOVA with Bonferroni's post hoc correction.

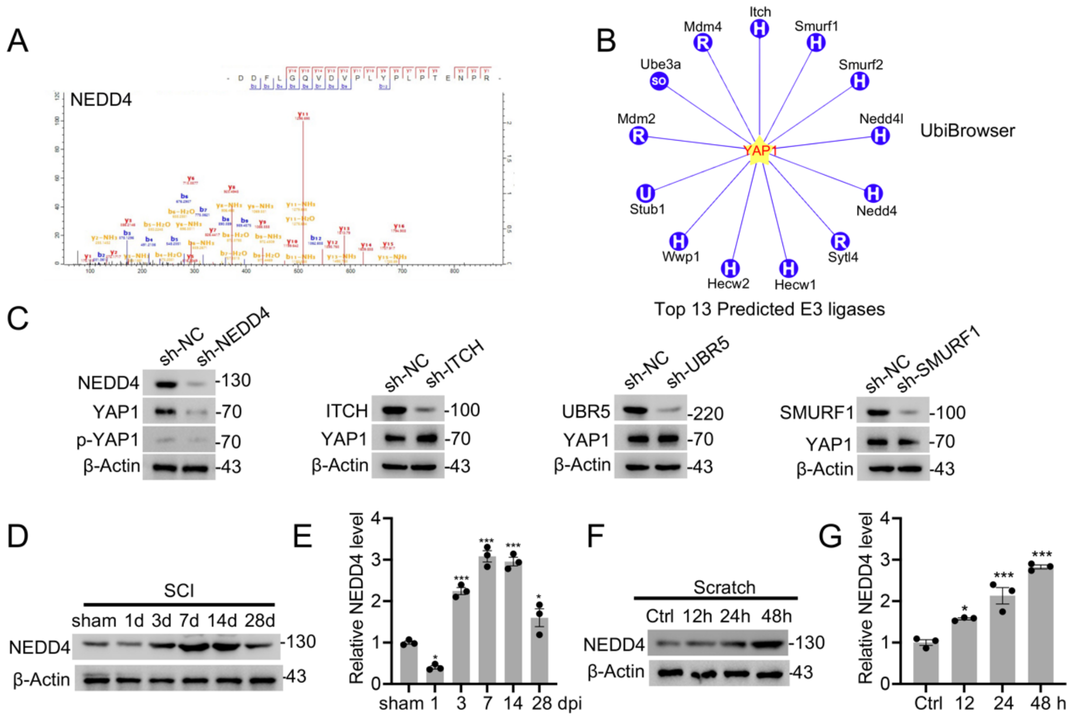

Fig.S2 Depletion of NEDD4 inhibits YAP1 protein expression. (A) IP/MS identified specific peptides of NEDD4 in immunoprecipitated YAP1 complexes in astrocytes. (B) UbiBrowser top13 predicated E3 ubiquitin ligases interact with YAP1. (C) Primary astrocytes were transfected with sh-NEDD4, sh-ITCH, sh-UBR5 and sh-SMURF1, and YAP1 and p-YAP1 expression testing. (D-E) Protein expression and quantification of NEDD4 of injured spinal cord at the indicated time after SCI (n=3). (F-G) Protein expression and quantification of YAP1 of astrocyte at the indicated time after *vitro* scratch injury(n=3). Statistical analysis for panels E and G was performed using one-way ANOVA with Bonferroni's post hoc correction.

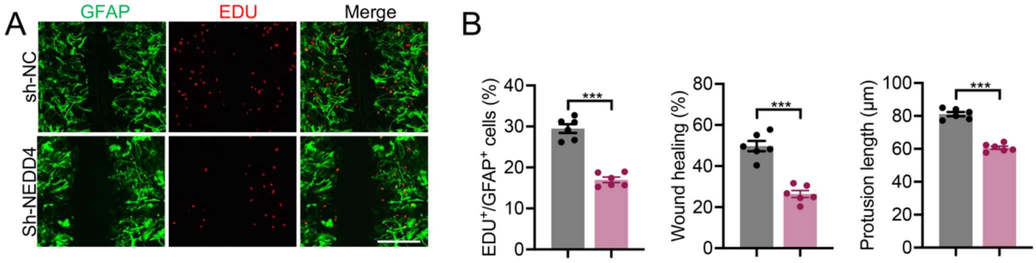

Fig. S3 Knockdown of NEDD4 attenuates proliferation and migration of reactive astrocytes. (A) Representative images of GFAP and EDU co-staining of primary astrocytes treated with sh-NC or sh-NEDD4 along with *vitro* stretch-injury after 24 hours (Scale bar = 50μm). (B) Quantification of EDU^{+}GFAP^{+}, wound healing and protrusion length. Statistical analysis for panel B was performed using a two-tailed unpaired Student's t-test.

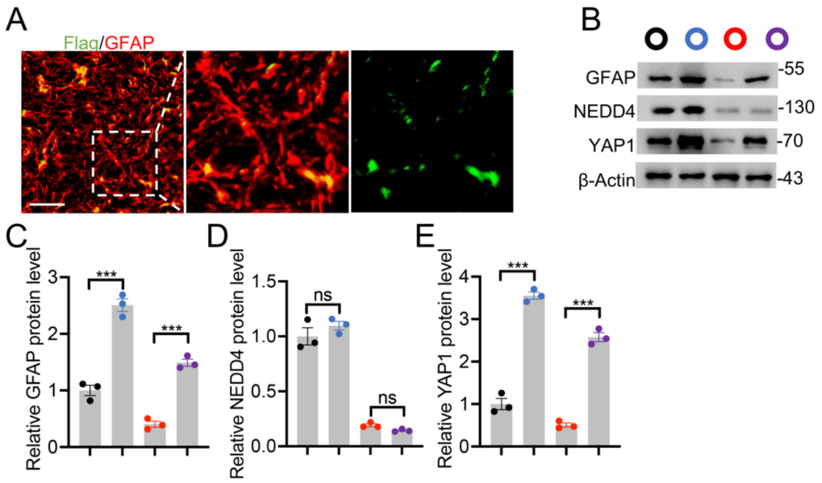

Fig. S4 Transfection efficiency of Flag-YAP1 in astrocytes. (A) Immunofluorescence analysis of Flag-YAP1 expression levels in astrocytes to assess viral transfection efficiency (Scale bar= 50μm). (B-E) Protein levels of GFAP, NEDD4 and YAP1 in indicated groups after SCI (n = 3) and quantification. Statistical analysis for panels C, D, and E was performed using a two-tailed unpaired Student's t-test.

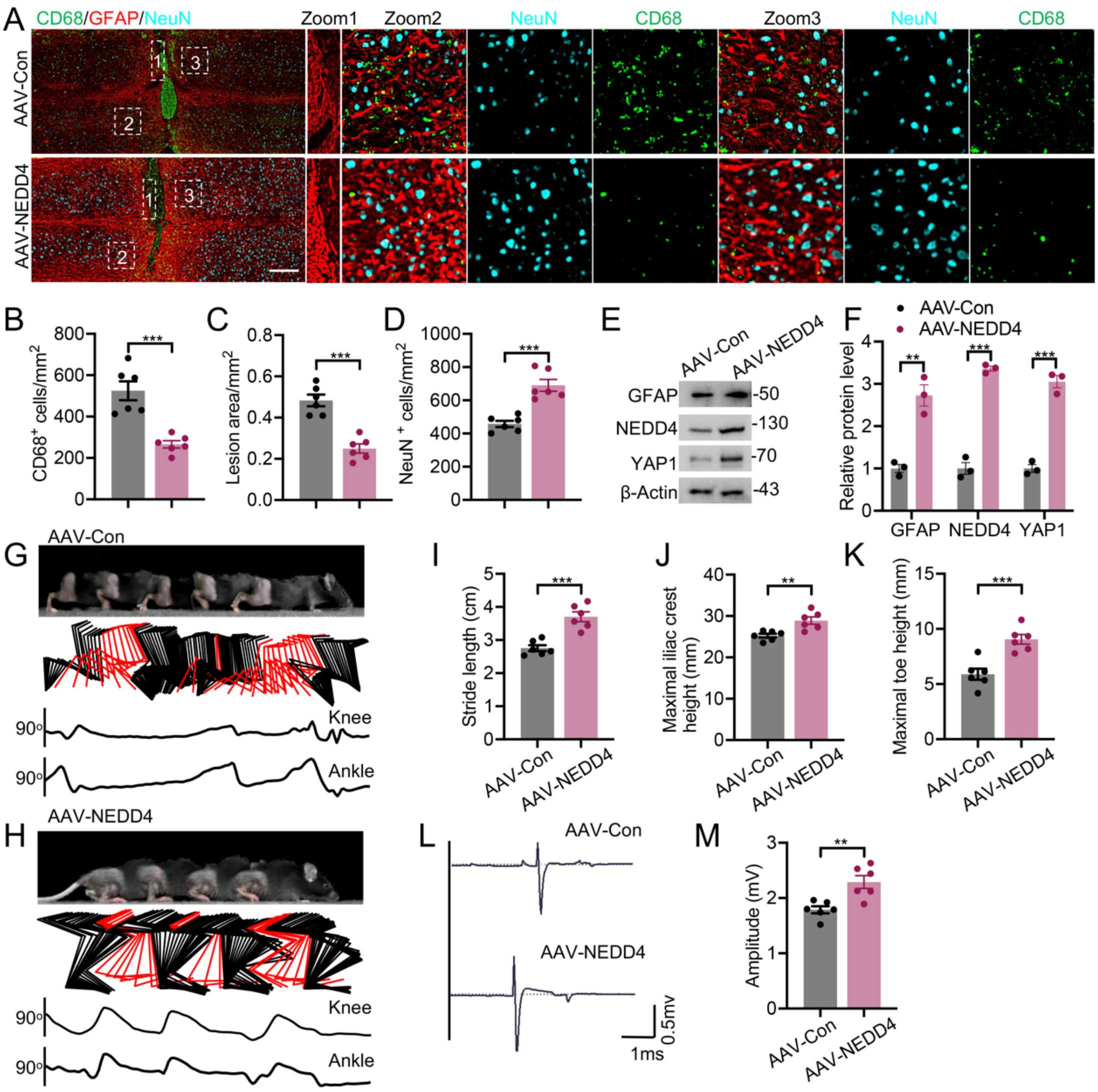

Fig. S5 Overexpression of NEDD4 facilitates functional restoration after SCI

(A-D) Representative confocal images showing co-staining of CD68, GFAP, and NeuN in spinal cord sections from the indicated groups at 14 dpi (A), accompanied by quantification of $CD68^+$ macrophages/microglia (B), lesion area (C), and neuronal number (D) (n = 6). (Scale bar = 200µm). (E-F) Indicated protein levels in primary astrocytes isolated from indicated groups and quantification (n = 3). (G-H) Chronophotographs of indicated groups at 28 dpi together with color-coded stick-figure representations of hindlimb movements, plotted joint angle excursions of the knee and ankle. (I-K) Measurements of stride length (I), maximal iliac crest height (J) and maximal toe elevation under indicated condition (K). (n = 6). (L-M) Representative EMG recordings of the gastrocnemius muscle, and their quantification in mice at 28 dpi (n = 6). Statistical analysis for panels B, C, E, F, I, J, K, and M was performed using

a two-tailed unpaired Student's t-test.
